# Examining potential confounding factors in gene expression analysis of human saliva and identifying potential housekeeping genes

**DOI:** 10.1038/s41598-022-05670-5

**Published:** 2022-02-10

**Authors:** P. Ostheim, S. W. Alemu, A. Tichý, I. Sirak, M. Davidkova, M. Markova Stastna, G. Kultova, S. Schuele, T. Paunesku, G. Woloschak, S. A. Ghandhi, S. A. Amundson, M. Haimerl, C. Stroszczynski, M. Port, M. Abend

**Affiliations:** 1 Bundeswehr Institute of Radiobiology affiliated to the University of Ulm, Neuherbergstr. 11, 80937 Munich, Germany; 2 Department of Radiobiology, Faculty of Military Health Sciences in Hradec Kralove, University of Defence in Brno, Brno, Czech Republic; 3 Biomedical Research Centre, University Hospital, Hradec Králové, Czech Republic; 4 Department of Oncology and Radiotherapy, University Hospital and Medical Faculty in Hradec Kralove, Hradec Králové, Czech Republic; 5 Department of Radiation Dosimetry, Nuclear Physics Institute of the Czech Academy of Sciences, Prague, Czech Republic; 6 Institute for Hematology and Blood Transfusion, Hospital Na Bulovce, Prague, Czech Republic; 7 Department of Radiation Oncology, Northwestern University, Chicago, IL 60611 USA; 8 Center for Radiological Research, Columbia University Irving Medical Center, New York, NY 10032 USA; 9 Department of Radiology, University Hospital Regensburg, Regensburg, Germany

**Keywords:** Molecular biology, Transcriptomics, Gene expression analysis, Reverse transcription polymerase chain reaction

## Abstract

Isolation of RNA from whole saliva, a non-invasive and easily accessible biofluid that is an attractive alternative to blood for high-throughput biodosimetry of radiological/nuclear victims might be of clinical significance for prediction and diagnosis of disease. In a previous analysis of 12 human samples we identified two challenges to measuring gene expression from total RNA: (1) the fraction of human RNA in whole saliva was low and (2) the bacterial contamination was overwhelming. To overcome these challenges, we performed selective cDNA synthesis for human RNA species only by employing poly(A)+-tail primers followed by qRT-PCR. In the current study, this approach was independently validated on 91 samples from 61 healthy donors. Additionally, we used the ratio of human to bacterial RNA to adjust the input RNA to include equal amounts of human RNA across all samples before cDNA synthesis, which then ensured comparable analysis using the same base human input material. Furthermore, we examined relative levels of ten known housekeeping genes, and assessed inter- and intra-individual differences in 61 salivary RNA isolates, while considering effects of demographical factors (e.g. sex, age), epidemiological factors comprising social habits (e.g. alcohol, cigarette consumption), oral hygiene (e.g. flossing, mouthwash), previous radiological diagnostic procedures (e.g. number of CT-scans) and saliva collection time (circadian periodic). Total human RNA amounts appeared significantly associated with age only (*P* ≤ 0.02). None of the chosen housekeeping genes showed significant circadian periodicity and either did not associate or were weakly associated with the 24 confounders examined, with one exception, 60% of genes were altered by mouthwash. *ATP6, ACTB* and *B2M* represented genes with the highest mean baseline expression (Ct-values ≤ 30) and were detected in all samples. Combining these housekeeping genes for normalization purposes did not decrease inter-individual variance, but increased the robustness. In summary, our work addresses critical confounders and provides important information for the successful examination of gene expression in human whole saliva.

## Introduction

Over the last two decades, saliva has become of increased interest as an easily accessible and non-invasive source of human biomarkers. Besides DNA, proteins and various metabolites, RNA (mRNA and miRNA species) has also been shown to be a promising marker in all tissues and body fluids^1–4^. Because saliva is derived from several tissue sources and also contains large amounts of total RNA it is one of the most attractive diagnostic, prognostic, and monitoring tools for both systemic and oral diseases^5–7^. As such, saliva has been shown to contain RNA biomarkers for prediction and diagnosis of several diseases especially of the oral cavity such as oral cancer^8–10^ and disorders of the salivary glands^11,12^. Saliva represents information from several bodily sources, including blood, because saliva is a plasma ultra-filtrate. This means that most compounds found in blood are also in saliva, leading to the aphorism that saliva is a “mirror of the body”^13,14^.

Also, collection of saliva samples represents an easy, cheap, non-invasive alternative to blood collection. In a previous study, we identified two problematic issues not coherently described before when working with human whole saliva: (1) the fraction of human RNA in whole saliva was low and (2) the bacterial contamination was overwhelming^15^. The previous study was based on 12 samples and served as an initial “proof-of-concept” study. Following this, in the current work, we have modified the methodology to (1) select only human RNA during cDNA synthesis by targeting the poly(A)+-tailed mRNA and (2) introduced pre-amplification of human RNA before qRT-PCR^15^. The combination of these two important modifications to our protocols resulted in sufficient amounts of high quality and quantity human RNA. In this context, we also discussed numerous advantages of collecting whole saliva as a source for RNA biomarkers instead of focusing on salivary supernatant^15^.

**Figure 1:**
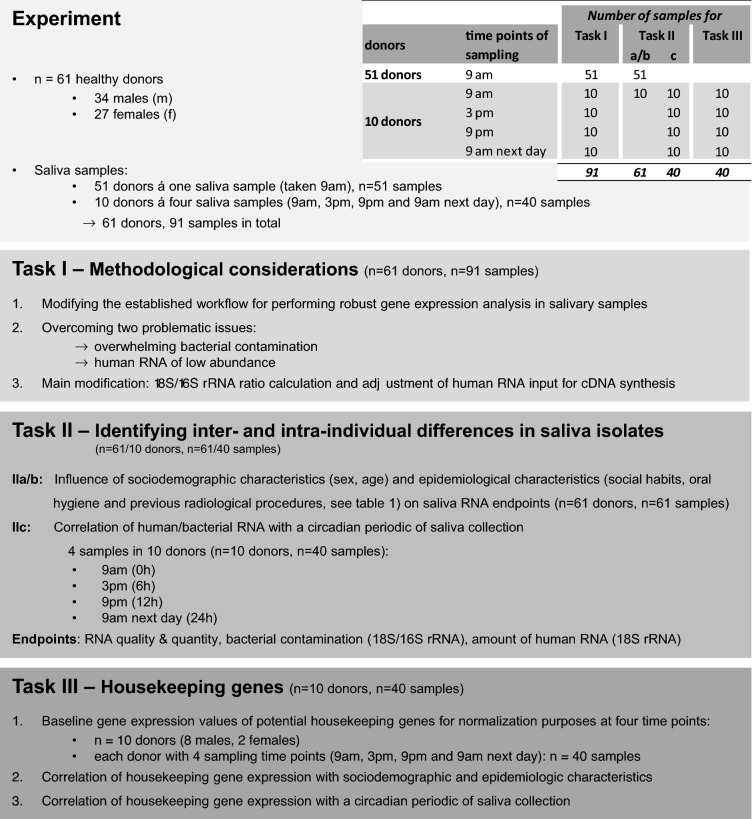
Overview of the samples, study design and tasks I–III description. The inserted table shows the number of samples used in the different tasks as well as the time points of sampling.

Not being a sterile medium like blood, saliva can be affected by many confounding variables, which then influence the quality and quantity of RNA that can be isolated from human whole saliva. The aim of this manuscript is to examine if there are any other prerequisites that have to be considered for satisfying results in this approach e.g. the optimal saliva sampling time or the impact of potential confounders such as demographics (e.g. sex, age) or epidemiological factors comprising social habits (e.g. alcohol, cigarette consumption), oral hygiene (e.g. flossing, mouthwash) or previous radiological diagnostic procedures (e.g. number of computed tomography/CT-scans). In the current exploratory study, we addressed the following aspects/tasks (Fig. 1): (I) For methodological reasons, we applied the previously described workflow^15^ using 91 instead of 12 samples (for validation purposes) and introduced the adaption of human RNA input for cDNA-synthesis to ensure equal human RNA amounts and improved comparability among different samples. (II) We examined inter- and intra-individual differences in salivary isolates considering potential demographic and epidemiological confounding factors (n = 24) as well as different saliva collection time points. (III) Human 18S rRNA, as a commonly known housekeeping gene, cannot be used for normalization purposes in gene expression analysis in the current application due to its lack of a poly-A-tail^15^. We examined baseline gene expression values of ten commonly used housekeeping genes in human whole saliva to accomplish the above mentioned tasks and present our analysis here.

## Materials and methods

### Sample collection

Whole saliva samples were collected using ORAgene®RNA (RE-100, catalog number: RE-100) vial collection kits from DNA Genotek according to the manufacturer’s instructions (DNA Genotek Inc., Kanata, Ontario, Canada). The kit is an all-in-one system for the collection, stabilization and transportation of RNA from saliva. Unstimulated whole saliva was collected from 61 healthy donors (27 females, 34 males, average age 38.5 ± 16.4 years, Fig. 1). The following exclusion criteria were applied: age below 18 years old, donors with history of immunodeficiency, autoimmune disorders, viral hepatitis, HIV infection, current or previous cancer, current oral problems (infection). From all donors, whole saliva was collected from 9 to 11 am and then preserved after having been shaken vigorously in the vial. Additionally, in 10 out of 61 donors, whole saliva samples were collected at 9 am, 3 pm, 9 pm and 9 am again the next day. After completing normal oral hygiene, donors were not allowed to eat or smoke 2 h prior to collection or to drink at least 1 h prior to collection. Samples were stored at room temperature overnight and placed in a freezer (− 20 °C) for storage. All samples were anonymized and obtained with informed consent from the donors. Sampling was carried out in accordance with the institutional guidelines and regulations. At the time of sampling, donors were asked to fill in a questionnaire (supplemental Figure 1) about demographics (e.g. sex, age), social habits (e.g. alcohol, smoking), oral hygiene (e.g. flossing, mouth wash), and previous radiological diagnostic procedures (e.g. number of CT-scans).

### RNA extraction

Total RNA comprising a mixture of human and bacterial RNA, was isolated from whole saliva samples following a combination of the ORAgene® RNA purification protocol^16^ and the mirVana™ kit protocol (Invitrogen™, ThermoFisher Scientific, Carlsbad, CA 92008; USA/Life Technologies, Darmstadt, Germany) as described in detail elsewhere^15^. In brief, the samples were heated at 50 °C (1 h), three aliquots (of 1000 µl) were generated, incubated at 90 °C (15 min), cooled to room temperature, 40 µl ORAgene® neutralizer solution (1/25 of total volume) was added, incubated on ice, centrifuged at 13,000*g* (3 min) and the cell-free clear supernatant was collected for further processing. At this step, we switched to the mirVana™ kit protocol^17^ by adding the Lysis/Binding Solution. With the mirVana™ kit, total RNA, including human and bacterial RNA species, was isolated by combining a Phenol–Chloroform RNA precipitation with further processing using silica membranes. After several washing procedures to purify RNA from other residual debris, DNA residuals were digested on the membrane (RNAse-free DNAse Set, Qiagen, Hilden, Germany). RNA was eluted with 100 µl RNAse free water in a collection tube and the aliquots were pooled for each sample. In order to increase the input RNA amount for downstream gene expression analysis, samples were steamed at 45 °C for 90 min followed by elution with 30 µl of RNase free water before freezing at − 20 °C.

Quality and quantity of isolated total RNA were measured spectrophotometrically using NanoDrop™ One Microvolume UV–Vis spectrophotometer (NanoDrop, PeqLab Biotechnology, Erlangen, Germany). RNA integrity was assessed by the 2100 Agilent Bioanalyzer (Life Science Group, Penzberg, Germany) and DNA contamination was controlled by conventional PCR using actin primers.

### Conventional cDNA synthesis—high-capacity cDNA reverse transcription kit

For analyzing gene expression of human rRNA (18S) and pan-bacterial rRNA (16S, see below), total salivary RNA was converted into complementary DNA (cDNA) via reverse transcription using the High-capacity cDNA reverse transcription kit^18^ (Applied Biosystems™, Life Technologies, Darmstadt, Germany). The amount of total RNA input was always determined to 500 ng measured by NanoDrop™ One. After reverse transcription, cDNA was diluted to a concentration of 0.01 ng/10 µl, which was used for qRT-PCR detection of 16S and 18S rRNA.

### Adjustment of human RNA input for cDNA synthesis

**Figure 2:**
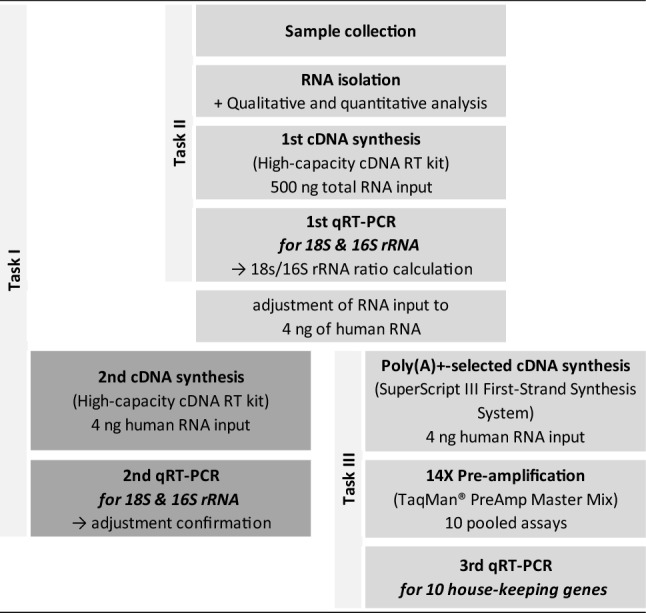
The flow chart displays the different steps (rows) in gene expression analysis including our modified workflow, the tasks, the required kits and the detour for adjustment of human RNA input as well as its confirmation (boxes in darker grey). The boxes in brighter grey depict the advanced methodological workflow for gene expression analysis in whole saliva samples.

A main modification of the previously described workflow^15^ was the adaption of human RNA input for cDNA synthesis with the SuperScript® III First-Strand Synthesis System (Fig. 2). As NanoDrop™ spectrophotometer only provides total RNA values comprising an unknown mixture of human and bacterial RNA species, we calculated the ratio of the detected raw Ct-values (threshold cycles) of human rRNA (18S) to pan-bacterial rRNA (16S, high-capacity cDNA reverse transcription kit cDNA synthesis followed by 1st qRT-PCR), regarding rRNA as representative for all human and bacterial RNA specimens. The ratio was determined by calculating the relative ratio of 18S rRNA to 16S rRNA for each sample individually as follows:

$$ {\text{Fold}}\,{\text{change}} = 2^{{ \wedge ({\text{Ct}}18{\text{S}}\,{\text{rRNA}} - {\text{Ct}}16{\text{S}}\,{\text{rRNA}})}} $$

Using the generated ratio, a RNA input of 4 ng total human RNA was determined individually for each sample for a second cDNA synthesis followed by 2nd qRT-PCR (confirmation for methodological reasons in this context, Fig. 2 boxes in darker grey).

### Modified cDNA synthesis—SuperScript III First-Strand Synthesis System

In order to subsequently perform gene expression analysis of human origin, only eukaryotic messenger RNA (mRNA) was reverse transcribed to cDNA by using the SuperScript® III First-Strand Synthesis System with Oligo (dT)_20_ primers. As a result, pan-bacterial RNA and other human RNA species were excluded from further processing, minimizing the inhibition effects of those RNA species on the following reactions. According to the kit description, the amount of starting material can vary from 1 pg to 5 μg of RNA and the maximum input volume of RNA is 8 µl^19^. The amount of human RNA input was set to 4 ng per reaction. Using the concentration from repeated NanoDrop™ measurements and the calculated 18S/16S ratio in each sample, the corresponding amount of measured total RNA input was calculated, conforming to the maximum input volume of 8 µl. The RT was performed according to the manufacturer’s instructions^19^.

### Pre-amplification—TaqMan® PreAmp Master Mix

To detect low abundance mRNA species, pre-amplification was required. We used TaqMan® PreAmp Master Mix (Thermo Fisher Scientific Baltics UAB, Vilnius, Lithuania) to increase the amount of specific cDNA targets, synthesized with the SuperScript® III First-Strand Synthesis System. According to the manufacturer, pre-amplification with this kit is linear when a minimum amount of cDNA molecules is present (minimum of 1–250 ng and Ct-values without pre-amplification should be < 35) and multiplex amplification can be performed by pooling up to 100 TaqMan® Gene Expression Assays. Pre-amplification was performed according to the TaqMan® PreAmp master mix kit protocol^20^. In the present work, 10 different TaqMan® Gene Expression Assays (*ACTB*, Hs01060665_g1; *B2M*, Hs00187842_m1; *GUSB*, Hs00939627_m1; *MT-ATP6*, Hs02596862_g1; *PGK1*, Hs00943178_g1; *PP1A*, Hs99999904_m1; *RPL13A*, Hs04194366_g1; *RPLP0*, Hs02992885_s1; *TBP*, Hs00427620_m1; *YWHAZ*, Hs01122445_g1) were utilized and pooled to enable the multiplex amplification of specific cDNA targets. Those are commonly used house-keeping genes already employed in other experimental set ups (www.genomics-online.com).

### Real-time quantitative reverse transcription polymerase chain reaction (qRT-PCR)

Using different sets of primers, two kinds of cDNAs were utilized for qRT-PCR: for human (18S rRNA, Hs99999901_g1) and pan-bacterial (16S rRNA, Ba04230899_s1) primer probe designs, cDNA from High-capacity cDNA reverse transcription kit was used, whereas for the primer probe designs representing the potential house-keeping genes (*ACTB*, *B2M*, *GUSB*, *MT-ATP6*, *PGK1*, *PP1A*, *RPL13A*, *RPLP0*, *TBP*, *YWHAZ*), SuperScript™ III First-Strand Synthesis SuperMix synthesized, i.e. human cDNA with and without 14× pre-amplification was used for detection of each of these genes in each sample. The qRT-PCR reaction contained TaqMan® Universal PCR Master Mix and one of the inventoried TaqMan® Gene Expression Assays for separate detection of transcripts. All measurements were run in duplicate, using a 96-well-format TaqMan® qRT-PCR platform and the QuantStudio™ 12 K Flex Real-Time PCR System.

### Statistical analysis

Descriptive statistics (n, mean, standard deviation, minimum [min], maximum [max]) were calculated for continuous variables such as gene expression data and age. Frequency tables of categorical data were examined for statistical differences using the chi-square test for equal proportion. Comparisons of categorical variables with gene expression values were performed using the non-parametric Kruskal–Wallis (KW) test. We assessed the assumptions of normality (Kolmogorov–Smirnov) and boxcox transformed the continuous variable “age”. All calculations were performed using SAS (release 9.4, Cary NC, USA). Graphical presentations were performed using Sigma Plot 14 (Jandel Scientific, Erkrath, Germany).

### Data availability and approval statement

The merged set of raw data is provided within supplemental Table 2. The qRT-PCR related measurements (e.g. RNA quantity/quality and TaqMan® qRT-PCR) were performed according to the standard operating procedures implemented in our laboratory in 2008 when the Bundeswehr Institute of Radiobiology became DIN-certified by TÜV Süd München, Germany (DIN EN ISO 9001/2008). All samples and data was processed anonymously without exception and only for this specific purpose. All data is handled according the European General Data Protection Regulation. Data will be deleted after 10 years. Due to the minimal-invasive collection and the fully anonymized processing of the samples, the local ethical commission (Ethics committee, Bayerische Landesärztekammer, Munich, Germany) decided that experiments can be performed in agreement with ethical standards and do not require an additional approval.

## Results

### Methodological considerations

#### RNA isolation

From 91 samples an average of 93.3 µg (SD ± 141.7) total RNA per 2 ml saliva could be isolated. Moreover, high purity RNA with OD_260/280_ ratios at a mean of 2.1 was isolated from saliva. A mean RNA integrity number (RIN) of 5.9 (SD ± 1.4) was detected for saliva samples and all saliva samples showed gel-like image bands of human 28S and 18S rRNA that did not provide any indications for severe degradation. No DNA contamination could be detected by beta actin PCR in all samples (data not shown).

#### Ratio of human to bacterial RNA

**Figure 3:**
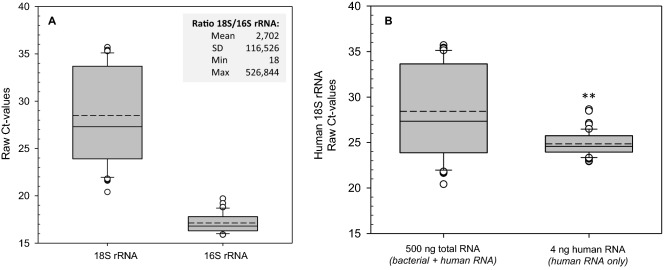
The box plots in (**A**) display the human 18S rRNA and bacterial 16S rRNA raw Ct values (threshold cycles) for all whole saliva samples (n = 91). Dashed lines represent the mean, solid lines the median and dots the outliers. The input amount for cDNA synthesis for each sample was 0.5 µg. The inserted table shows the calculated ratio between raw Ct values of human 18S rRNA and bacterial 16S rRNA and provides descriptive statistics: mean, minimum [min], maximum [max], standard deviation [stdev] and standard error of the mean [sem]. The box plots in (**B**) represent the human 18S rRNA raw Ct-values before and after adjustments accounting for input differences from left to right. The left part shows Ct values from 1st qRT-PCR performed using cDNA with an input of 500 ng total RNA (bacterial and human RNA) and the right boxplot the corresponding results when taking 4 ng of human RNA (calculated via the 18S/16S-ratio together with total RNA concentration values measured). Asterisks (**) refer to a *P* value < 0.001 using 500 ng total RNA measurements as reference.

Human 18S rRNA and pan-bacterial 16S rRNA raw Ct-values were measured from whole saliva via qRT-PCR following a first cDNA-synthesis with the High-capacity Kit (total RNA input of 500 ng). A mean human 18S rRNA raw Ct-value of 28.5 (SD ± 4.9, min 20.4, max 35.7) and a mean bacterial 16S rRNA raw Ct-value of 17.1 (SD ± 1.04, min 15.9, max 19.7) implied on average about 2,702 (2^28.5–17.1^) times more bacterial RNA copy numbers relative to human RNA in whole saliva (Fig. 3A). This means that for each copy of a human gene, on average 2,702-times more copies of bacterial genes can be found in the samples. Human 18S rRNA raw Ct values showed a broad variance with almost 10 Ct values in the 50% interquartile range, indicating about 1000-fold differences in RNA copy numbers (Fig. 3A).

#### Adjustment of human RNA input for cDNA-synthesis and its confirmation

Using the generated ratio (ratio = 2^^(Ct18S rRNA—Ct16S rRNA)^), a defined amount of human RNA (4 ng) was reverse transcribed in a second cDNA synthesis, again using High-capacity cDNA reverse transcription kit, followed by qRT-PCR analysis for detection of human 18S rRNA and bacterial 16S rRNA Ct-values (Fig. 3B). The 50% interquartile range of human 18S rRNA quantification in 51 saliva samples changed significantly (*P* < 0.001) from 7 Ct values of unadjusted input RNA (comprising an unknown mixture of human and bacterial RNA) to 1.5 Ct values after considering the degree of bacterial contamination and adjusting for it.

### Identifying inter- and intra-individual differences in saliva RNA isolates

#### Sociodemographic and epidemiological characteristic of donors

The 61 healthy donors comprised almost equal proportions of females (44.3%) and males (55.7%, Table 1). Donors were mostly Caucasians (77.1%) and about half of them were aged 19–30 years at time of saliva collection. Only 18% smoked over at least 5 years and another 16% of former smoker smoked at least 2 years and stopped smoking about a year ago. Alcohol consumption and diet was reported by 41% and 28%, respectively. Most (83.6%) of the donors brushed their teeth at least twice a day and about half of them used flossing and about one-third mouthwash. Braces (3.3%) and dentures (11.5%) were reported less frequently and oral problems like periodontitis in 23%. Acute and chronic diseases such as rheumatism or disease of the thyroid gland were reported in 6.6% and 16.4%, respectively. Radiological examinations during the last six months (mainly X-rays and CT-scans) were reported in 18% and none of our donors received radiotherapy.

**Table 1:** The table summarizes the characteristics of all 61 donors such as demographics (e.g. sex, age), social habits (e.g. alcohol, cigarette consumption), oral hygiene (e.g. flossing, mouth wash), acute/chronic diseases and previous radiological procedures (e.g. number of CT-scans).

Parameter	categories	Number (n = 61)	percent	*P* value
Demographic characteristics				
Sex	Female	27	44.3	
	Male	34	55.7	0.4
Race	Caucasian	47	77.1	
	Other	14	23.0	< 0.0001
Age (years)	≥ 19–30	30	49.2	
	> 30–50	15	24.6	
	> 50	16	26.2	< 0.0001
	n	61		
	Mean	38.5		
	Stdev	16.5		
	sem	2.1		
	Min	19.0		
	Max	75.0		
Social habits				
Current smoker	Yes	11	18.0	
	No	50	82.0	< 0.0001
Cigarettes (#/day)	11–20	4	6.6	
	21–30	4	6.6	
	31–40	3	4.9	
	None	50	81.9	< 0.0001
Duration of smoking (years)	≥ 5	11	18.0	
	None	50	82.0	< 0.0001
Former smoker	Yes	10	16.4	
	No	44	72.1	
	Unknown	7	11.5	< 0.0001
Duration former smoker (years)	≥ 2	10	16.4	
	None	51	83.6	< 0.0001
Duration since stopped smoking (years)	≥ 1	10	16.4	
	None	51	83.6	< 0.0001
Alcohol consumption (#/week)	< 1	25	41.0	
	> 1	28	45.9	
	None	8	13.1	0.003
Diet	Yes	17	27.9	
	No	43	70.5	
	Others	1	1.6	< 0.0001
Oral hygiene				
Oral hygiene (#/day)	≤ 1x/d	10	16.4	
	≥ 2x/d	51	83.6	< 0.0001
Flossing	Yes	30	49.2	0.90
	No	31	50.8	
Flossing use (#/month)	≥ 1–10	11	18.0	
	> 10	18	29.5	
	None	32	52.5	0.04
Mouth wash	Yes	19	31.2	
	No	42	68.9	0.003
Mouth wash (#/month)	≥ 4–16	8	13.1	
	≥ 30	11	18.0	
	None	42	68.9	< 0.0001
Braces use	Yes	2	3.3	
	No	59	96.7	< 0.0001
Denture use	Yes	7	11.5	
	No	54	88.5	< 0.0001
Oral problems	Yes	14	23.0	
	No	47	77.1	< 0.0001
Acute/chronic diseases				
Acute disease	Yes	4	6.6	
	No	57	93.4	< 0.0001
Chronic disease	Yes	10	16.4	
	No	51	83.6	< 0.0001
Diagnostic and therapeutic procedures				
Radiological examinations (last 6 months)	Ever	11	18.0	
	Never	50	82.0	< 0.0001
Type of radiological examinations (last 6 months, multiple entries)	X-ray	11	15.7	
	CT-scan	6	8.6	
	Nuclear medicine	1	1.4	
	PET/SPECT	2	2.9	
	None	50	71.4	< 0.0001
Radiotherapy	No	61	100	n.a

Shown are the numbers per group with descriptive statistics where appropriate (n, mean, minimum [min], maximum [max], standard deviation [stdev] and standard error of the mean [sem]), the percentage per category and the corresponding *P* values (chi-square test).

#### Correlation of human/bacterial RNA with sociodemographic and epidemiological characteristics

Total RNA concentration increased almost three-fold with higher alcohol consumption (data not shown). Only human RNA (18S rRNA) amount as well as the ratio of 18S/16S rRNA appeared significantly associated with age on a categorical (*P* = 0.07, 0.04) or linear scale (*P* = 0.02, 0.02), which was not shown for bacterial RNA (16S rRNA). We built two groups based on relatively high (18S rRNA Ct value ≤ 30) and relatively low (18S RNA Ct value > 30) amounts of human RNA. Within the younger age group (< 30 years) the mean human RNA amount (18S rRNA as representative) was 676-fold higher between both human 18S rRNA groups, but neither sociodemographic nor epidemiological parameters appeared significantly associated (data not shown). In the older (> 30 years old) compared to younger donor group (≤ 30 years old), examinations regarding the high yield human 18S rRNA group showed significantly more alcohol consumption per week (*P* = 0.012), higher frequency of former smoker (*P* = 0.0058), radiological examinations (*P* = 0.012) and dentures (*P* = 0.032).

#### Correlation of human/bacterial RNA with a circadian periodic saliva collection

**Figure 4:**
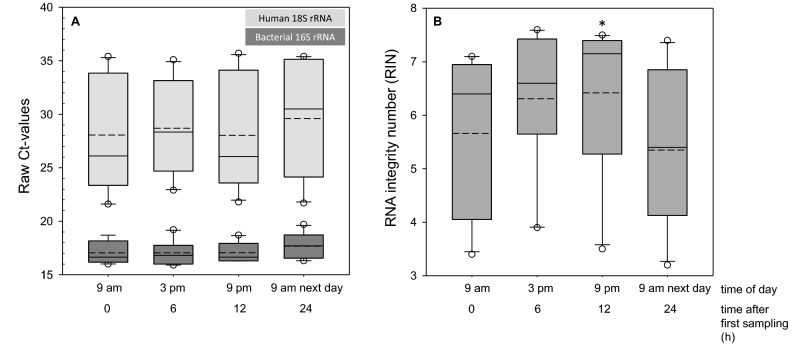
Box plots in (**A**) show the human 18S rRNA and bacterial 16S rRNA raw Ct-values for whole saliva samples (total n = 40) per time point (each n = 10: 9 am—0 h; 3 pm—6 h; 9 pm—12 h; 9 am next day—24 h). The input amount for cDNA synthesis for each sample was 0.5 µg. The box plots in (**B**) represent the quality of isolated RNA using RNA integrity numbers (RIN) for saliva samples (total n = 40) per time point (each n = 10). Dashed lines represent the mean, solid lines the median and dots the outliers. The asterisk (*) refers to a *P* value < 0.05 using 9 am measurements as the reference.

Among all time points, the amount of bacterial RNA was constantly high (mean raw Ct values of bacterial 16S rRNA were 17.0–17.7) with a small variance (SD raw Ct values of bacterial 16S rRNA 0.9–1.1 over all time points) whereas the amount of human RNA was lower (mean raw Ct values of human 18S rRNA 28.0–29.6) with a large variance (SD raw Ct values of human 18S rRNA 4.0–5.2 over all time points, Fig. 4A). The intra-individual variance of human 18S rRNA was very high in most of the samples with a SD of up to 5.8 among the time points. In donor 1, we observed a minimum human 18S rRNA raw Ct value of 21.7 (9 am the next day) and a maximum 18S rRNA raw Ct value of 35.1 (3 pm), resulting in a delta Ct value of more than 13 or in other words more than 8,000-fold difference in human RNA amount (data not shown). Relative to the earliest sampling time at 9 am, slightly increased median RIN values and about two-fold decreased interquartile ranges were observed at 3 and 9 pm (Fig. 4B, *P* = 0.022), but distribution of RIN values at 9 am of the following day were similar to the 9 am values of the previous day (Fig. 4B).

### Identifying inter- and intra-individual differences in housekeeping genes

#### Baseline gene expression values of housekeeping genes

**Figure 5:**
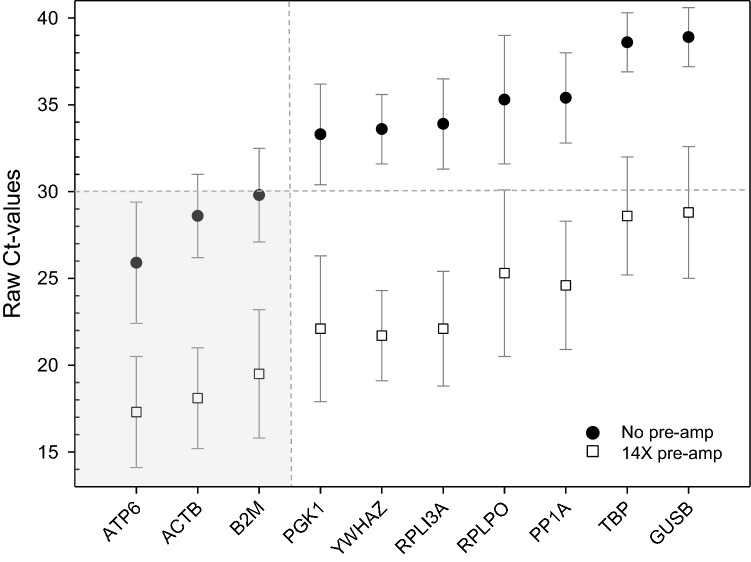
The graph represents raw Ct values (threshold cycles) of potential housekeeping genes (n = 10) analyzed for the 40 samples from 10 donors (four time points per donor), arranged in ascending order of raw unamplified Ct values. For each gene, the Ct values measured via qRT-PCR with cDNA without pre-amplification as well as after 14× pre-amplification is shown. Symbols represent geometric mean values and error bars reflect the standard error of mean per gene. Vertical and horizontal grey dashed lines show the cut-off (mean Ct ≤ 30). Three of the genes (*ACTB*, *ATP6* and *B2M*, highlighted in grey area) showed un-amplified Ct-values < 30 indicating that no pre-amplification for adequate detection will be required.

**Figure 6:**
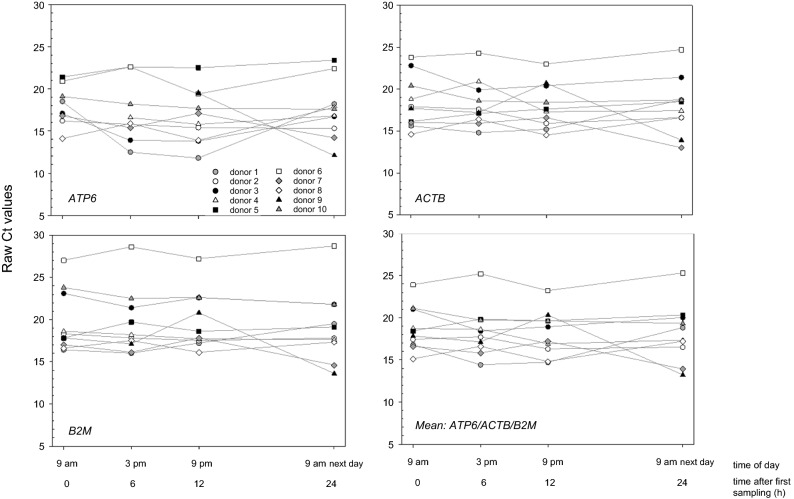
Raw Ct values (threshold cycles) of three candidate house-keeping genes (*ACTB*, *ATP6* and *B2M*) as well as a combination of them (arithmetic mean) are depicted over time (four time point each: 9 am—0 h; 3 pm—6 h; 9 pm—12 h; 9 am next day—24 h) for each donor. They were fulfilling the criteria for being an appropriate housekeeping gene in this context.

Among ten examined housekeeping genes, seven (*GUSB*, *PGK1*, *PP1A*, *RPL13A*, *RPLP0*, *TBP*, *YWHAZ)* revealed baseline levels > 30 Ct-values without pre-amplification (Fig. 5). Three genes revealed Ct-values < 30 without pre-amplification, namely *ATP6* (mean raw Ct value = 25.9), *ACTB* (mean raw Ct value = 28.6) and *B2M* (mean raw Ct value = 29.8) indicating high expression levels and detection in all samples (Figs. 5, 6, supplemental Table 1, supplemental Table 2).

#### Correlation of housekeeping gene expression with sociodemographic and epidemiological characteristics

Eight of ten housekeeping genes showed a weak (*P* = 0.02–0.05) but significant association of altered gene expression and oral hygiene like mouth wash and flossing (Table 2). Further weak associations were found for alcohol consumption with *PGK1* (*P* = 0.03), age with *RPLPO* (*P* = 0.034) and radiological examination during the last 6 months with *ATP6* gene expression (*P* = 0.046). Only *TBP* showed no significant association with sociodemographic and epidemiological characteristics (Table 2).

**Table 2:** Overview of the housekeeping gene expression results (raw Ct values either without pre-amplification or after 14× pre-amplification) and the significant correlations with sociodemographic and epidemiological characteristics.

	raw Ct value						
	n	Mean	stdev	sem	Min	Max	*P* value
*PGK1*—no preamp							
Alcohol consumption per week							
None	1	31.5			31.5	31.5	
< 1	4	34.9	0.6	0.3	34.4	35.7	
> 1	5	32.8	1.9	0.8	29.6	34.1	0.03
*PGK1*—14 × preamp							
Mouth wash (#/month)							
None	5	21.5	0.5	0.2	21	22.3	
≥ 4–16	2	16.6	3.3	2.3	14.3	19	
≥ 30	3	27.2	3.9	2.2	23.8	31.4	0.022
*ACTB*—no preamp							
Flossing							
Yes	7	29.3	2.2	0.8	27.5	33.6	
No	3	26.8	0.4	0.2	26.4	27.2	0.02
Flossing (#/month)							
None	3	26.8	0.4	0.2	26.4	27.2	
≥ 1–10	5	28.5	1.3	0.6	27.5	30.3	
> 10	2	31.2	3.4	2.4	28.8	33.6	0.04
*ACTB*—14 × preamp							
Flossing (#/month)							
None	3	16	1.6	0.9	14.6	17.7	
≥ 1–10	5	17.8	1.9	0.8	16	20.4	
> 10	2	23.3	0.7	0.5	22.8	23.8	0.047
Mouth wash (#/month)							
None	5	17.2	1.4	0.6	15.6	18.8	
≥ 4–16	2	15.4	1.1	0.8	14.6	16.1	
≥ 30	3	22.3	1.7	1	20.4	23.8	0.041
*B2M*—no preamp							
Mouth wash							
Yes	5	31	1.7	0.8	29.3	33.7	
No	5	28.2	0.8	0.4	27.1	29.4	0.016
Mouth wash (#/month)							
None	5	28.2	0.8	0.4	27.1	29.4	
≥ 4–16	2	30.3	1.4	1	29.3	31.3	
≥ 30	3	31.4	2	1.2	29.9	33.7	0.0497
*B2M*—14× preamp							
Flossing							
Yes	5	31	1.7	0.8	29.3	33.7	
No	5	28.2	0.8	0.4	27.1	29.4	0.02
Flossing (#/month)							
None	3	16.9	0.7	0.4	16.4	17.8	
≥ 1–10	5	19.1	2.7	1.2	17	23.8	
> 10	2	25.1	2.8	2	23.1	27	0.048
*RPLPO*—no preamp							
Mouth wash							
Yes	4	33.3	2.3	1.2	30.7	36.3	
No	3	37.8	1.4	0.8	36.4	39.1	0.034
Age (years)							
≥ 19–30	3	37.8	1.4	0.8	36.4	39.1	
> 30–50	4	33.3	2.3	1.2	30.7	36.3	
> 50	0						0.034
*GUSB*—14× preamp							
Mouth wash							
Yes	3	31.5	3.1	1.8	28.1	34.3	
No	5	26.9	1	0.4	25.2	27.9	0.025
*PP1A*—14× preamp							
Mouth wash							
Yes	5	28.4	4	1.8	25.3	34.7	
No	5	23.3	1.9	0.9	20.8	25.8	0.016
Mouth wash (#/month)							
None	5	23.3	1.9	0.9	20.8	25.8	
≥ 4–16	2	25.6	0.5	0.4	25.3	26	
≥ 30	3	30.2	4.3	2.5	26.1	34.7	0.031
YWHAZ—14× preamp							
Mouth wash							
Yes	5	23.8	3.1	1.4	20.1	27.4	
No	5	20	0.9	0.4	19	21.2	0.047
Mouth wash (#/month)							
None	5	20	0.9	0.4	19	21.2	
≥ 4–16	2	20.5	0.6	0.4	20.1	21	
≥ 30	3	26	1.3	0.8	24.8	27.4	0.049
*ATP6*—no preamp							
Oral problems							
Yes	2	30.3	4.8	3.4	26.9	33.6	
No	8	28.1	1.3	0.5	26.4	30.3	0.037
*ATP6*—14× preamp							
Radiological examination (last 6 months)							
Never	6	18.9	1.9	0.8	16.8	21.4	
Ever	2	15.1	1.5	1.1	14.1	16.2	0.046
*RPLI3A*—14× preamp							
Mouth wash (#/month)							
None	5	23.1	1.8	0.8	20.9	25.5	
≥ 4–16	2	19.2	1.3	0.9	18.3	20.1	
≥ 30	3	26.8	4	2.3	23.1	31.1	0.047

Provided are numbers (n) per category and corresponding descriptive statistics: mean, minimum [min], maximum [max], standard deviation [stdev] and standard error of the mean [sem]. Comparisons of categorical variables with gene expression values were performed using the non-parametrical Kruskal–Wallis (KW) test.

#### Correlation of housekeeping gene expression with a circadian periodic of saliva collection

None of the housekeeping genes revealed significant gene expression changes associated with the time of saliva sampling, but different patterns in gene expression changes over time of sampling were observed for individuals (Fig. 6). For example, we found a decrease of normalized Ct values among all genes in donor 9 at 9 am on the next day, indicating a pattern, caused by methodological reasons. A combination of *ATP6, ACTB* and *B2M* housekeeping genes (mean of Ct values) did not reduce inter-individual variability significantly (Fig. 6).

## Discussion

The search for simple less-invasive sampling methods plays an important role for high-throughput diagnostic tests like for victims of radio/nuclear incidents. Besides whole blood, saliva, a non-invasive easily-accessible biofluid, has been shown to contain mRNA biomarkers for prediction and diagnosis of several diseases^21,22^. Examinations in this regard are challenged, because our previous studies indicate that most of the isolated RNA originates from the oral microbiome, thus, reducing the amount of isolated human RNA considerably. We previously modified the methodology to better analyse the low abundance of the human RNA fraction from whole saliva^15^.

In the current study, we confirmed previous results increasing the sample size from 12 to 91 whole saliva samples. Furthermore, we modified the previously described workflow to ensure equal human RNA input for cDNA-synthesis as a prerequisite for comparability among samples when performing quantitative RT-PCR. In addition to normalization using a housekeeping gene, this normalization step via RNA quantification proved to be a robust method when measuring RNA expression between samples^23^. Spectrophotometrically, only total RNA (non-specific human and bacterial) can be measured. Measurement of total RNA quantity is relatively uninformative considering the high and inhomogeneous bacterial contamination of saliva samples. To resolve this issue, we relatively quantified human RNA using 18S rRNA as surrogate and bacterial contamination using 16S rRNA as surrogate, and introduced a correction factor for the same starting material (human RNA amount) for downstream cDNA-synthesis. By performing a second cDNA-synthesis with the High-capacity Kit (followed by qRT-PCR with detection of human 18S rRNA and bacterial 16S rRNA) we confirmed that we were able to adjust the amount of human RNA input for downstream cDNA synthesis with the SuperScript® III First-Strand Synthesis System (amount of starting material: 1 pg–5 µg^19^) to equal amounts.

In the current study we also examined factors with potential impact on RNA quantity and quality. Those included different collection time points for saliva sampling or extraneous factors, demographic and epidemiological. Concerning circadian periodic rhythmicity, published protocols from other research groups recommend highest yields of RNA as well as best quality when sampling between 9 and 11 am ^9,24–28^. Our study indicated no significant differences in RNA amounts between the collection time points, but intra-individual differences were high (> 1000-fold). Also, RIN values slightly improved at later (3 pm and 9 pm) relative to early collection time points (9 am), indicating that saliva samples can be collected during the whole day, thus, widening the applicability of this approach, e.g. for clinical use.

We did not observe any significant differences concerning sociodemographic and epidemiological characteristics that would explain the observed magnitude of inter-individual variance of human RNA yields. Furthermore, the differences in amounts of human RNA (raw Ct value for 18S rRNA was ≤ 30) between the samples could not be explained by sociodemographic or epidemiological characteristics. In this study, the addressed sociodemographic and epidemiological conditions seemed to be of minor relevance for interpretation of saliva gene expression results.

Human 18S rRNA, as a commonly known housekeeping gene, cannot be used as a normalizer in gene expression analysis in the current application due to the lack of a poly(A)+-tail ^15^. We examined the baseline gene expression values of ten commonly used housekeeping genes. These genes appeared not or only weakly altered by sociodemographic or epidemiological factors which adds to their robustness. *ATP6, ACTB* and *B2M* appeared most suitable, because sufficiently high copy numbers ensured detection in all samples indicating methodological robustness. However, inter-individual differences in gene expression and certain time points were found in several donors. These patterns occurred among all genes, suggesting they may be caused by methodological reasons. Combining all three housekeeping genes in our study did not reduce the variance significantly, but normalization based on more than one reference gene has been increasingly suggested by others^29–31^. Certainly, combining *ATP6, ACTB* and *B2M* as housekeeping genes for expression studies using human saliva will increase the robustness and, therefore, would be suggested. Nevertheless, planned future studies on saliva samples from irradiated donors will finally show whether radiation impacts these three identified genes, which would render them unsuitable as housekeeping genes. The applicability of suggested housekeeping genes has to be proven in future independent studies.

Finally, some limitations of this manuscript need to be considered. The major limitation lies in the fact that epidemiological data was gathered from the healthy donors by anamnesis. For example, the oral health status was self-reported and not confirmed by medical examination.

Nevertheless, a main strength of this study is that it represents a comprehensive examination of different facets when working with human whole saliva. The enhancement of the methodology and the proper examination of potential confounders like the influence of sociodemographic and epidemiologic characteristics that could potentially influence salivary isolates are completely novel findings, not described before in the literature. Considering saliva as an emerging source of body fluid for gene expression examinations underlines the importance of those findings. A key strength of the present study was the sample size: 91 samples from 61 donors in total are remarkable numbers considering molecular biological studies. These numbers and the numerous endpoints in this study are sufficient for creating reliable hypotheses.

In summary, we (I) improved the comparability of gene expression measurements among different saliva samples, (II) demonstrated that quality and quantity of RNA isolates is highly robust considering potential confounding factors such as demographics/epidemiologic and the saliva sampling time, making the approach of saliva collection even more attractive for further biomarker studies and (III) identified a set of potential housekeeping genes (*ATP6, ACTB* and *B2M*) and suggested their combination to increase robustness of saliva-based gene expression studies.

## Supplementary Information

Supplementary Figure 1.

Supplementary Table 1.

Supplementary Table 2.
