# Supplementary material for: Examining potential confounding factors in gene expression analysis of human saliva and identifying potential housekeeping genes
Source: Sci Rep. 2022 Feb 10;12:2312. doi: 10.1038/s41598-022-05670-5 (PMC8831573; doi:10.1038/s41598-022-05670-5)
Supplement: Supplementary file 1 — Supplementary Figure 1. [file 41598_2022_5670_MOESM1_ESM.pdf]

Sample ID______00_____

1. **How old are you? _______**

2. **Gender**

   ☐ **Female**

   ☐ **Male**

3. **Race/Ethnicity?**

   ☐ **Caucasian (light skin, European origin)**

   ☐ **Asian**

   ☐ **Hispanic**

   ☐ **African**

   ☐ **Mixed Race**

   ☐ **Others**

4. **Do you currently smoke? If yes, how many cigarettes per day so you usually smoke?**

   ☐ **1-10**

   ☐ **11-20**

   ☐ **21-30**

   ☐ **31-40**

   ☐ **41 and more**

   For how long have you smoked? _______________

5. **Are you a former smoker? If yes, for how long did you smoke?_______________**

   How long has it been since you quit smoking? _______________

6. **How often do you drink alcohol (beer, wine and others) in a week?**

   ☐ **Never**

   ☐ **Less than 1x per week**

   ☐ **1-2x per week**

   ☐ **3-6x per week**

   ☐ **7-10x per week**

   ☐ **11-12x per week**

   ☐ **More than 12x per week**

7. What is your diet?

- ☐ **Vegan/Vegetarian**
- ☐ **High meat diet**
- ☐ **No diet**
- ☐ **Others ________________________**

8. Have you ever undergone radiological imaging in the last 6 months? If yes, which ones

- ☐ **X-ray**
- ☐ **PET/SPECT**
- ☐ **CT-scan**
- ☐ **Angiography**
- ☐ **Nuclear medicine**

9. Do you currently have or previously had any form of acute/chronic disease (Example: Diabetes, Cancer)?____________________

- ☐ **Yes, I am suffering from an acute illness _____________________________**
- ☐ **No, I am not suffering from an acute illness**
- ☐ **Yes, I am suffering from a chronic illness _____________________________**
- ☐ **No, I am not suffering from a chronic illness**

If yes, did you receive radiotherapy as part of your treatment? ______________

10. How often do your brush your teeth?

- ☐ **Daily**
- ☐ **Once every 2 days**
- ☐ **Once every 3 days**

If daily, how many times do you brush your teeth?

- ☐ **Once a day**
- ☐ **Twice a day**
- ☐ **More**

11. Do you use braces? (Yes / No) or dentures (Yes / No)

12. Do you currently floss or use mouthwash? If yes, please state and clarify how often?

☐ **Floss** **How often?**______________

☐ **Mouthwash** **How often?**______________

13. Have you currently ☐

or previously ☐

Had an oral disease or any oral associated problems?
(Parotitis, gingivitis, permanent wound)

____

Date ______________ **Time**______________

Supplemental figure 1
Shown is the questionnaire (English version), which was completed by each donor covering demographic characteristics, social habits, lifestyle and other factors such as oral hygiene, radiological imaging, chronic and acute diseases and the prevalence of oral diseases.
