# Supplementary material for: Examining potential confounding factors in gene expression analysis of human saliva and identifying potential housekeeping genes
Source: Sci Rep. 2022 Feb 10;12:2312. doi: 10.1038/s41598-022-05670-5 (PMC8831573; doi:10.1038/s41598-022-05670-5)
Supplement: Supplementary file 2 — Supplementary Table 1. [file 41598_2022_5670_MOESM2_ESM.pdf]

Supplemental table 1
Ostheim et al.

Gene	Preamplifi-cation		Descriptive statistic of Ct values				
		n	mean	stdev	sem	min	max
16S rRNA		40	**17.2**	1.1	0.2	15.9	19.7
18S rRNA		40	**28.6**	5	0.8	21.6	35.7
ACTB	No	40	**28.6**	2.4	0.4	24.6	33.6
	14X	40	**18.1**	2.9	0.5	13	24.7
ATP6	No	39	**25.9**	3.5	0.6	17.8	35.8
	14X	36	**17.3**	3.2	0.5	11.8	23.4
B2M	No	40	**29.8**	2.7	0.4	24.6	35.5
	14X	40	**19.5**	3.7	0.6	13.6	28.7
GUSB	No	22	**38.9**	1.7	0.4	35.7	42
	14X	35	**28.8**	3.8	0.6	21.5	41.1
PGK1	No	40	**33.3**	2.9	0.5	25.7	38.5
	14X	40	**22.1**	4.2	0.7	14.3	31.4
PP1A	No	36	**35.4**	2.6	0.4	28.9	41.3
	14X	40	**24.6**	3.7	0.6	16.8	34.7
RPLI3A	No	40	**33.9**	2.6	0.4	28.2	38.3
	14X	40	**22.1**	3.3	0.5	15.6	31.1
RPLPO	No	35	**35.3**	3.7	0.6	27.1	41.5
	14X	40	**25.3**	4.8	0.8	15.4	36.7
TBP	No	22	**38.6**	1.7	0.4	34.4	42.3
	14X	30	**28.6**	3.4	0.6	21.7	35.8
YWHAZ	No	39	**33.6**	2	0.3	30	38.4
	14X	40	**21.7**	2.6	0.4	16.8	27.7

Supplemental table 1
Displayed are descriptive statistics (mean, minimum [min], maximum [max], standard deviation [stdev] and standard error of the mean [sem]) of the raw Ct values for human 18S rRNA and bacterial 16S rRNA as well as for the potential housekeeping genes (n=10). Furthermore, shown are the number (n) of detected amplification plots (ideally n=40 from 40 samples) per gene.
