# Supplementary material for: Examining potential confounding factors in gene expression analysis of human saliva and identifying potential housekeeping genes
Source: Sci Rep. 2022 Feb 10;12:2312. doi: 10.1038/s41598-022-05670-5 (PMC8831573; doi:10.1038/s41598-022-05670-5)
Supplement: Supplementary file 3 — Supplementary Table 2. [file 41598_2022_5670_MOESM3_ESM.pdf]

Supplemental table 2

Ostheim et al.

	Questionnaire - results																					
Sample ID	Age	Gender	Race	Current smoker	Number of cigarettes per day	Duration of smoking	Former smoker	Duration of former smoking	Duration since quitting smoking	Diet	Radiological imaging in the last 6 months	Acute disease	Chronic disease	Radiotherapy	Tooth brushing frequency	Use of braces	Use of dentures	Flossing	Flossing frequency per month	Mouth wash	Mouth wash frequency per month	Oral diseases
**1**	20	1	1	1	1	5	0			3	0	0	0	0	5	0	0	1	12	1	4	0
**2**	60	1	1	1	1	45	0			3	1	0	1	0	5	0	0	0		1	30	0
**3**	74	2	1	0	0		1	30	18	2	2	1	0	0	5	0	1	0		1	30	0
**4**	55	1	1	0	0		0			3	2	1	0	0	6	0	0	1	30	0		0
**5**	62	2	1	0	0		1	10	40	2	1	0	0	0	6	0	0	0		0		0
**6**	25	1	2	0	0		0			2	0	0	0	0	6	0	0	0		0		0
**7**	25	1	5	0	0		0			3	0	0	1	0	4	0	0	1	15	0		0
**8**	30	2	1	0	0		0			3	0	0	0	0	5	0	0	0		0		0
**9**	27	1	1	0	0		0			3	0	0	0	0	5	0	0	1	60	0		0
**10**	26	1	1	0	0		0			3	0	0	0	0	5	0	0	1	4	0		0
**11**	70	1	1	0	0		0			2	0	0	0	0	5	0	0	0		1	60	3
**12**	60	1	1	0	0		0			3	1	0	1	0	5	0	0	1	4	0		3
**13**	66	2	1	0	0		0			3	0	0	0	0	6	0	0	1	30	0		3
**14**	75	2	1	0	0		1	55	1	2	2	1	0	0	5	0	1	0		0		0
**15**	27	2	1	0	0		0			2	0	0	1	0	6	0	0	1	8	1	4	0
**16**	27	1	2	0	0		0			1	0	0	0	0	4	0	0	0		0		0
**17**	57	1	1	0	0		1	6	37	3	3	0	0	0	5	0	0	1	30	0		0
**18**	25	1	1	0	0		0			3	0	0	0	0	5	0	0	0		0		0
**19**	27	1	1	1	2	10	0			3	0	0	0	0	5	0	0	1	15	0		0
**20**	27	2	1	0	0		0			3	0	0	0	0	5	0	0	1	60	1	60	0
**21**	26	1	1	0	0		0			3	0	0	1	0	5	0	0	1	4	0		0
**22**	33	2	1	1	3	14	0			3	0	0	0	0	5	0	0	1		1	30	0
**23**	45	1	1	1	2	25	0			3	0	0	0	0	2	0	0	0		1	16	1+2
**24**	33	2	1	0	0		1	2	14	1	0	0	0	0	5	0	0	0		0		2
**25**	38	1	1	0	0		0			3	0	0	0	0	5	0	0	1	10	1	4	3
**26**	60	1	1	1	2	45	0			3	0	0	1	0	5	0	0	1	30	0		0
**27**	64	2	1	1	1	45	0			3	0	0	1	0	5	0	1	1	30	0		0
**28**	61	1	1	0	0		1	13	27	3	0	0	1	0	6	0	1	1	30	1	30	2
**29**	22	1	2	0	0		0			2	0	0	0	0	5	0	0	0		0		0
**30**	24	1	1	1	2	10	0			1	0	0	0	0	5	0	0	1	30	1	30	0
**31**	28	2	1	1	3	14	0			2	0	0	0	0	5	0	0	0		0		0
**32**	26	2	4	0	0		0			2	0	0	0	0	4	0	0	0		0		0
**33**	31	2	1	0	0		0			3	0	0	0	0	5	0	0	1	30	1	30	0
**34**	44	2	4	0	0		0			2	0	0	0	0	5	0	0	0		0		0
**35**	26	2	2	0	0		0			3	0	0	0	0	4	0	0	0		0		0
**36**	24	1	2	0	0		0			1	0	0	0	0	4	0	1	0		0		0
**37**	21	1	1	0	0		0			3	0	0	0	0	2	0	0	1	15	1	15	2
**38**	25	2	1	0	0		0			3	0	0	0	0	4	1	0	0		0		2
**39**	40	1	2	0	0		0			1	0	1	0	0	5	0	0	0		0		0
**40**	26	2	2	0	0		0			3	0	0	0	0	5	0	0	0		0		0
**41**	19	2	1	0	0		0			3	0	0	0	0	5	0	0	0		0		0
**42**	71	2	1	0	0		1	25	30	3	0	0	1	0	4	0	1	0		0		0
**43**	66	2	1	0	0		0			3	0	0	0	0	5	0	0	1	1	1	4	0
**44**	62	1	1	0	0		1	10	36	3	1	0	0	0	5	0	1	0		0		3
**45**	33	2	1	0	0		0			3	0	0	0	0	5	0	0	0		0		2
**46**	28	2	5	0	0		0			2	0	0	0	0	5	0	0	1	30	0		0
**47**	30	2	1	1	1	8	0			3	0	0	0	0	5	0	0	0		0		2
**48**	39	1	1	0	0		1	6	15	3	0	0	0	0	5	0	0	0		0		0
**49**	49	2	1	0	0		1	20	6	3	0	0	0	0	5	0	0	1	30	0		0
**50**	27	2	1	0	0		0			3	0	0	0	0	5	0	0	0		0		0
**51**	32	2	1	1	3	8	0			3	1	0	0	0	4	0	0	0		0		0
**1.1**	27	2	1	0	0		0			2	0	0	0	0	5	0	0	0		0		2
**1.2**																						
**1.3**																						
**1.4**																						
**2.1**	29	2	1	0	0		0			3	1	0	0	0	5	1	0	1	4	0		0
**2.2**																						
**2.3**																						
**2.4**																						
**3.1**	32	2	1	0	0		0			4	0	0	0	0	5	0	0	1	15	1	30	2
**3.2**																						
**3.3**																						
**3.4**																						
**4.1**	24	2	1	0	0		0			3	0	0	0	0	5	0	0	1	8	0		0
**4.2**																						
**4.3**																						
**4.4**																						
**5.1**	36	2	1	0	0		0			3	0	0	0	0	5	0	0	1	4	1	8	0
**5.2**																						
**5.3**																						
**5.4**																						
**6.1**	35	2	6	0	0		0			3	0	0	0	0	5	0	0	1	30	1	30	0
**6.2**																						
**6.3**																						
**6.4**																						
**7.1**	25	1	4	0	0		0			3	0	0	0	0	5	0	0	1	1	0		0
**7.2**																						
**7.3**																						
**7.4**																						
**8.1**	38	1	6	0	0		0			3	1	0	0	0	5	0	0	0		1	8	0
**8.2**																						
**8.3**																						
**8.4**																						
**9.1**	58	2	1	0	0		0			3	0	0	1	0	5	0	0	0		0		0
**9.2**																						
**9.3**																						
**9.4**																						
**10.1**	29	2	1	0	0		0			3	0	0	0	0	5	0	0	1	4	1	30	0
**10.2**																						
**10.3**																						
**10.4**																						

	Quality & Quantity (after pooling 3 aliquots à 100µl = 300µl)					Bacterial contamination Ratio Calculation					
	Nano drop			Agilent	β-Actin-Gel						
Sample ID	260/280	RNA concentration (ng/µl)	RNA amount (µg/300µl)	RIN	DNA contamination	18S Ct mean	delta replicates	16S Ct mean	delta replicates	Fold Δ 18S-16S	2^Fold Δ 18S-16S
**1**	1.9	16.9	56.2	4.2	Negative	25.4	0.42	18.7	-0.08	6.7	103
**2**	1.7	7.7	25.8	8.5	Negative	35.1	0.44	19.2	0.00	15.8	58689
**3**	1.9	19.7	65.7	7.6	Negative	34.5	-0.96	18.3	0.01	16.2	73881
**4**	2.1	76.8	255.9	4.5	Negative	21.7	0.67	16.4	0.03	5.4	41
**5**	2.1	27.3	91.1	7.4	Negative	34.3	-1.87	16.0	-0.02	18.3	324790
**6**	2.0	12.7	42.3	7.7	Negative	33.1	-0.22	16.0	-0.01	17.1	139580
**7**	1.9	3.3	11.1	7.0	Negative	26.4	0.31	17.8	0.19	8.6	386
**8**	2.1	17.0	56.8	6.9	Negative	35.1	-0.52	19.7	0.06	15.4	44128
**9**	2.0	5.8	19.3	7.3	Negative	24.5	0.24	16.1	0.06	8.3	325
**10**	2.0	13.8	46.0	6.2	Negative	23.4	0.29	15.9	0.06	7.5	175
**11**	2.1	34.7	115.7	4.9	Negative	25.7	-0.08	16.3	-0.02	9.4	691
**12**	2.1	38.3	127.7	3.2	Negative	28.7	-0.11	18.7	0.28	9.9	976
**13**	2.2	119.3	397.8	4.0	Negative	23.9	0.06	16.3	0.02	7.5	184
**14**	2.1	37.3	124.3	6.3	Negative	27.3	0.29	17.3	-0.06	10.0	1018
**15**	2.0	12.2	40.6	6.2	Negative	23.5	0.17	16.3	0.02	7.2	152
**16**	2.1	11.4	38.0	4.1	Negative	27.0	0.09	18.8	0.04	8.1	282
**17**	2.1	61.7	205.7	6.0	Negative	33.7	0.76	16.2	-0.01	17.4	173912
**18**	2.1	24.2	80.7	5.6	Negative	28.1	0.35	17.4	0.07	10.7	1651
**19**	2.1	28.5	94.9	3.5	Negative	31.1	0.22	18.7	-0.09	12.4	5351
**20**	2.1	229.1	763.5	5.3	Negative	32.3	0.71	18.2	-0.03	14.1	17795
**21**	2.0	13.1	43.7	5.7	Negative	35.4	1.39	18.7	0.11	16.7	105527
**22**	2.0	7.7	25.5	3.0	Negative	33.3	0.15	18.8	-0.04	14.5	23661
**23**	2.1	14.9	49.7	9.1	Negative	35.7	0.51	17.3	-0.02	18.5	360270
**24**	2.0	7.4	24.7	6.6	Negative	24.6	0.53	17.8	0.10	6.9	118
**25**	1.9	6.7	22.3	6.3	Negative	26.8	0.40	18.0	0.13	8.7	430
**26**	2.0	12.0	39.9	4.6	Negative	28.6	0.02	17.1	-0.13	11.6	3004
**27**	2.1	16.4	54.6	6.1	Negative	24.0	0.35	16.8	-0.01	7.2	147
**28**	2.1	17.3	57.6	6.7	Negative	35.3	0.82	16.3	-0.01	19.0	526844
**29**	2.1	27.0	90.0	6.0	Negative	21.6	-0.35	16.8	-0.05	4.8	29
**30**	2..06	15.9	53.0	6.5	Negative	25.1	0.17	16.0	-0.04	9.1	556
**31**	2.1	19.3	64.4	6.0	Negative	23.6	-0.19	16.3	0.02	7.3	154
**32**	2.1	15.4	51.2	5.7	Negative	35.4	-0.52	17.6	0.25	17.8	226316
**33**	2.0	13.9	46.4	7.1	Negative	33.3	-0.94	17.1	0.12	16.3	78102
**34**	2.0	9.7	32.2	7.3	Negative	30.1	0.23	16.5	0.02	13.6	12580
**35**	2.1	44.5	148.3	7.2	Negative	21.8	0.06	16.5	-0.03	5.3	40
**36**	2.0	15.8	52.7	9.1	Negative	33.4	0.29	16.6	0.15	16.7	108988
**37**	2.1	38.8	129.2	6.1	Negative	21.7	0.05	16.5	0.00	5.2	36
**38**	2.2	115.8	386.1	4.1	Negative	22.9	0.35	16.2	0.07	6.7	104
**39**	2.0	16.2	54.0	6.4	Negative	34.0	0.82	16.3	-0.02	17.7	214543
**40**	2.1	43.1	143.7	4.4	Negative	22.7	-0.42	16.7	0.05	6.0	63
**41**	2.2	91.6	305.3	5.9	Negative	24.2	0.46	16.9	-0.02	7.4	164
**42**	2.1	32.0	106.5	5.9	Negative	20.4	-0.08	16.3	-0.08	4.1	18
**43**	2.1	81.8	272.7	5.4	Negative	35.1	1.64	17.3	0.10	17.8	227507
**44**	2.1	51.3	171.0	5.3	Negative	34.5	-0.46	16.2	0.00	18.2	307026
**45**	2.1	124.5	415.0	5.8	Negative	25.3	0.22	16.7	0.13	8.5	367
**46**	2.1	60.8	202.7	6.1	Negative	22.6	-0.36	15.9	0.03	6.7	107
**47**	2.1	57.9	193.1	5.5	Negative	23.2	-0.01	16.1	-0.07	7.1	135
**48**	2.2	251.6	838.5	3.2	Negative	27.3	0.16	17.3	-0.08	10.0	1002
**49**	2.2	76.0	253.4	4.5	Negative	27.8	0.01	16.4	0.13	11.4	2710
**50**	2.2	113.8	379.3	6.1	Negative	31.6	-0.15	16.9	0.01	14.8	28476
**51**	2.2	19.4	64.6	6.2	Negative	33.8	-0.03	15.9	0.03	17.9	244265
**1.1**	2.0	34.0	10.2	4.1	Negative	25.4	0.42	18.7	-0.08	6.7	103
**1.2**	2.0	56.5	17.0	7.4	Negative	35.1	0.44	19.2	0.00	15.8	58689
**1.3**	2.1	113.0	33.9	7.4	Negative	34.5	-0.96	18.3	0.01	16.2	73881
**1.4**	2.0	51.0	15.3	4.2	Negative	21.7	0.67	16.4	0.03	5.4	41
**2.1**	2.0	33.5	10.1	6.8	Negative	34.3	-1.87	16.0	-0.02	18.3	324790
**2.2**	2.0	82.4	24.7	7.0	Negative	33.1	-0.22	16.0	-0.01	17.1	139580
**2.3**	1.9	32.4	9.7	7.4	Negative	26.4	0.31	17.8	0.19	8.6	386
**2.4**	1.7	17.2	5.2	7.0	Negative	35.1	-0.52	19.7	0.06	15.4	44128
**3.1**	2.1	329.8	98.9	7.1	Negative	24.5	0.24	16.1	0.06	8.3	325
**3.2**	2.1	193.2	58.0	7.6	Negative	23.4	0.29	15.9	0.06	7.5	175
**3.3**	2.1	177.0	53.1	7.4	Negative	25.7	-0.08	16.3	-0.02	9.4	691
**3.4**	2.1	195.0	58.5	3.2	Negative	28.7	-0.11	18.7	0.28	9.9	976
**4.1**	2.2	399.8	119.9	6.2	Negative	23.9	0.06	16.3	0.02	7.5	184
**4.2**	2.1	169.6	50.9	6.2	Negative	27.3	0.29	17.3	-0.06	10.0	1018
**4.3**	2.0	45.7	13.7	7.5	Negative	23.5	0.17	16.3	0.02	7.2	152
**4.4**	2.0	77.9	23.4	7.4	Negative	27.0	0.09	18.8	0.04	8.1	282
**5.1**	2.1	117.6	35.3	6.6	Negative	33.7	0.76	16.2	-0.01	17.4	173912
**5.2**	2.0	67.6	20.3	6.3	Negative	28.1	0.35	17.4	0.07	10.7	1651
**5.3**	2.0	89.1	26.7	6.8	Negative	31.1	0.22	18.7	-0.09	12.4	5351
**5.4**	2.1	136.4	40.9	5.9	Negative	32.3	0.71	18.2	-0.03	14.1	17795
**6.1**	2.1	298.2	89.5	6.9	Negative	35.4	1.39	18.7	0.11	16.7	105527
**6.2**	2.1	201.1	60.3	6.8	Negative	33.3	0.15	18.8	-0.04	14.5	23661
**6.3**	2.0	68.2	20.5	3.5	Negative	35.7	0.51	17.3	-0.02	18.5	360270
**6.4**	2.1	131.6	39.5	6.8	Negative	24.6	0.53	17.8	0.10	6.9	118
**7.1**	1.9	16.9	5.1	4.5	Negative	26.8	0.40	18.0	0.13	8.7	430
**7.2**	2.0	19.0	5.7	6.4	Negative	28.6	0.02	17.1	-0.13	11.6	3004
**7.3**	1.8	9.3	2.8	7.2	Negative	24.0	0.35	16.8	-0.01	7.2	147
**7.4**	1.8	17.5	5.3	4.9	Negative	35.3	0.82	16.3	-0.01	19.0	526844
**8.1**	1.8	21.8	6.5	3.4	Negative	21.6	-0.35	16.8	-0.05	4.8	29
**8.2**	1.8	22.1	6.6	3.9	Negative	25.1	0.17	16.0	-0.04	9.1	556
**8.3**	1.8	23.2	7.0	4.3	Negative	23.6	-0.19	16.3	0.02	7.3	154
**8.4**	1.9	27.2	8.2	3.9	Negative	35.4	-0.52	17.6	0.25	17.8	226316
**9.1**	1.9	34.6	10.4	3.9	Negative	33.3	-0.94	17.1	0.12	16.3	78102
**9.2**	2.0	40.6	12.2	4.0	Negative	30.1	0.23	16.5	0.02	13.6	12580
**9.3**	1.7	12.7	3.8	5.6	Negative	21.8	0.06	16.5	-0.03	5.3	40
**9.4**	2.1	97.9	29.4	4.2	Negative	33.4	0.29	16.6	0.15	16.7	108988
**10.1**	1.7	9.3	2.8	7.1	Negative	21.7	0.05	16.5	0.00	5.2	36
**10.2**	1.9	17.1	5.1	7.5	Negative	22.9	0.35	16.2	0.07	6.7	104
**10.3**	1.8	21.1	6.3	7.1	Negative	34.0	0.82	16.3	-0.02	17.7	214543
**10.4**	1.9	16.9	5.1	6.0	Negative	22.7	-0.42	16.7	0.05	6.0	63

	Endogenous control genes																													
	ACTB			ATP6			B2M			GUSB			PGK1			PP1A			RPLI3A			RPLPO			TBP			YWHAZ		
Sample ID	No Pre-Amp	14X	Delta	No Pre-Amp	14X	Delta	No Pre-Amp	14X	Delta	No Pre-Amp	14X	Delta	No Pre-Amp	14X	Delta	No Pre-Amp	14X	Delta	No Pre-Amp	14X	Delta	No Pre-Amp	14X	Delta	No Pre-Amp	14X	Delta	No Pre-Amp	14X	Delta
**1.1**	26.9	15.6	11.3	30.5	18.5	12.0	28.0	16.4	11.6	39.2	27.2	12.0	34.1	21.0	13.2	36.0	25.8	10.2	38.3	25.5	12.8		30.4		38.5	27.3	11.2	34.6	21.2	13.4
**1.2**	26.6	14.8	11.8	24.5	12.5	12.1	29.4	16.0	13.4		26.7		31.7	19.0	12.7	32.6	18.4	14.1	29.2	16.3	12.9	28.6	16.5	12.1				33.8	20.4	13.5
**1.3**	26.1	15.2	10.8	22.2	11.8	10.4	29.9	17.2	12.6		27.3		30.0	17.8	12.2	31.4	19.3	12.1	28.2	15.6	12.6	27.1	15.4	11.7		26.7		32.8	20.4	12.4
**1.4**	30.4	18.7	11.7	29.9	18.2	11.7	32.1	19.5	12.6	41.7	29.7	12.0	37.2	23.9	13.3	40.3	25.8	14.5	36.5	23.6	12.8	36.9	24.2	12.7	41.1	29.8	11.3	34.9	21.5	13.4
**2.1**	27.5	17.9	9.6	22.3	16.2	6.1	28.4	18.3	10.1	37.8	27.1	10.7	32.7	21.0	11.7	34.1	22.1	12.0	34.5	22.4	12.1	39.1	26.7	12.3	38.0	26.5	11.5	32.0	19.4	12.6
**2.2**	26.5	17.6	8.9	22.1			26.9	17.8	9.1	37.5	26.0	11.5	32.3	20.8	11.6	33.6	21.7	11.8	34.1	21.8	12.2	38.4	26.7	11.7	37.9	25.9	12.0	31.9	19.3	12.6
**2.3**	26.5	15.9	10.5	23.9	15.4	8.5	27.8	17.5	10.3		26.9		33.2	20.9	12.3	34.3	22.2	12.1	34.2	22.0	12.3		27.7		36.9	26.5	10.5	31.7	19.0	12.7
**2.4**	27.7	16.6	11.1	23.6	15.3	8.3	27.6	17.8	9.8		27.2		34.1	22.3	11.9	33.3	21.6	11.7	32.9	20.6	12.3	37.4	26.7	10.6	36.9	26.5	10.5	32.2	19.7	12.5
**3.1**	33.6	22.8	10.9	35.8	17.1	18.7	33.7	23.1	10.6				29.6	23.8	5.8		34.7		34.1	23.1	11.0	33.5	23.6	10.0					25.6	
**3.2**	30.3	19.9	10.5	32.1	13.9	18.2	34.4	21.4	13.0		26.5		25.7	20.9	4.8	35.0	24.5	10.4	31.6	18.9	12.7	31.1	19.6	11.5				35.3	23.3	12.0
**3.3**	31.1	20.4	10.7	32.2	13.8	18.4	35.1	22.6	12.5				25.7	21.4	4.3		27.1		31.0	19.0	12.0	31.1	19.6	11.5				36.7	24.6	12.1
**3.4**	33.4	21.4	12.0		16.7		34.4	21.8	12.6		32.1		27.6	25.4	2.3		28.2		37.0	24.1	12.9	37.1	24.9	12.2		32.4		38.4	25.6	12.8
**4.1**	29.7	18.8	10.9	21.5			28.2	18.6	9.6	36.8	25.2	11.6	33.6	22.3	11.4	32.7	20.8	11.8	32.8	20.9	11.9	36.4	25.3	11.1	38.8	27.0	11.8	32.8	20.6	12.2
**4.2**	32.2	20.9	11.3	22.6	16.6	6.0	27.7	18.2	9.5	38.8	27.0	11.9	35.9	24.7	11.2	34.4	22.3	12.1	34.1	22.2	11.9	39.9	27.4	12.5		29.8		35.0	22.4	12.6
**4.3**	27.4	17.3	10.1	22.4	15.8	6.6	27.7	17.7	10.0	38.2	25.9	12.3	32.6	20.6	12.0	33.1	21.3	11.8	33.3	20.9	12.3	37.7	26.3	11.3	36.9	26.3	10.6	31.7	18.9	12.8
**4.4**	26.6	17.4	9.1	23.5	16.8	6.7	26.5	17.6	8.9	37.5	24.5	13.0	32.7	20.9	11.8	33.0	21.4	11.6	33.5	21.5	12.1	37.3	26.8	10.6	39.9	26.4	13.5	32.0	19.7	12.3
**5.1**	27.7	16.1	11.6	26.0	21.4	4.6	31.3	17.8	13.5		28.1		34.0	14.3	19.7	36.5	26.0	10.5	33.0	20.1	12.9	32.6	20.0	12.6		27.2		32.5	20.1	12.4
**5.2**	28.9	17.1	11.8	27.4	22.6	4.8	32.4	19.7	12.7	37.5	30.7	6.8	35.5	15.3	20.2	37.6	26.2	11.4	32.9	20.5	12.4	33.8	21.4	12.4	37.1			32.8	19.7	13.1
**5.3**	29.2	17.6	11.7	24.3	22.5	1.7	31.7	18.6	13.1		28.5		34.5	14.6	19.8	38.2	26.4	11.8	33.1	20.4	12.6	33.0	21.4	11.6		28.7		32.0	19.6	12.4
**5.4**	30.6	18.5	12.1	26.0	23.4	2.6	31.7	19.1	12.6	39.1	29.6	9.5	36.0	15.5	20.5	38.8	26.0	12.8	35.4	22.6	12.8	35.9	23.7	12.2	39.6	28.5	11.0	35.0	22.6	12.4
**6.1**	28.8	23.8	5.0	23.9	20.9	3.0	29.9	27.0	2.8	37.3	34.3	3.0	34.7	31.4	3.3	37.2	29.8	7.3	36.9	31.1	5.8	36.3	32.0	4.4		35.8		35.1	27.4	7.7
**6.2**	33.0	24.3	8.7	29.0	22.6	6.4	35.5	28.6	6.9	41.3	36.4	5.0	37.7	31.2	6.5	41.3	31.7	9.6	36.0	26.8	9.2	35.7	27.5	8.2				38.4	27.7	10.7
**6.3**	32.9	23.0	10.0	26.3	19.4	7.0	34.2	27.2	7.0		36.2		38.5	30.8	7.7	40.5	31.3	9.1	37.5	26.8	10.7	35.7	26.9	8.8		35.8		35.7	23.7	12.0
**6.4**	27.4	24.7	2.7	23.0	22.4	0.6	29.4	28.7	0.7		41.1		32.8	31.3	1.5	36.5	31.8	4.7	30.6	26.8	3.8	30.3	27.4	2.9				31.6	25.6	6.0
**7.1**	27.6	16.0	11.6	28.7	16.8	11.9	29.4	17.0	12.4		27.9		34.6	21.5	13.1	37.0	24.5	12.4	38.1	24.3	13.8	37.8	27.8	10.1	39.2	27.3	11.9	32.5	19.0	13.5
**7.2**	26.0	15.9	10.1	23.3	15.4	7.9	27.3	16.1	11.1				32.9	20.2	12.7	33.0	20.5	12.5	33.1	20.4	12.7	37.9	25.5	12.4	37.2	24.6	12.6	31.7	18.5	13.2
**7.3**	29.1	16.6	12.5	29.7	17.1	12.6	30.3	17.8	12.5				34.1	22.3	11.8		23.4		34.6	21.5	13.1	34.7	22.2	12.5				34.6	22.9	11.8
**7.4**	25.4	13.0	12.4	26.1	14.2	11.9	27.6	14.6	12.9		25.0		30.9	20.2	10.7	32.9	22.3	10.6	30.7	19.0	11.6	31.4	21.0	10.4				32.2	19.2	13.0
**8.1**	27.2	14.6	12.6	26.3	14.1	12.2	29.3	16.6	12.7	36.4			31.5	19.0	12.5	36.2	25.3	11.0	31.2	18.3	12.9	30.7	18.5	12.2				32.8	21.0	11.9
**8.2**	28.5	16.4	12.1	27.4	15.9	11.5	30.0	17.5	12.6		28.6		36.2	22.6	13.7	37.1	24.2	12.9	37.3	23.9	13.4	38.8	25.6	13.2		27.4		34.6	21.5	13.2
**8.3**	26.5	14.5	12.1	25.3	13.9	11.4	28.3	16.1	12.2		28.1		31.7	19.3	12.4	35.2	23.6	11.6	29.9	17.4	12.5	29.9	18.0	11.9		27.2		33.3	20.5	12.8
**8.4**	27.9	16.6	11.3	30.0	17.8	12.2	29.3	17.3	12.0	40.2	28.6	11.6	35.7	22.6	13.1	37.9	25.1	12.8	36.6	23.3	13.2	36.4	24.1	12.3	39.0	28.3	10.8	34.6	21.5	13.1
**9.1**	26.4	17.7	8.7	25.3			27.1	17.8	9.3	39.5	27.0	12.5	34.4	21.6	12.8	35.7	23.0	12.7	35.5	22.4	13.1		32.6		38.7	26.5	12.2	32.5	19.7	12.8
**9.2**	25.9	17.2	8.8	24.1			26.6	17.1	9.5	39.1	25.9	13.2	33.0	20.3	12.7	34.1	21.5	12.7	33.4	20.5	12.8	40.9	30.2	10.7	38.4	25.4	13.0	31.8	19.0	12.8
**9.3**	32.0	20.7	11.3	29.6	19.5	10.1	32.8	20.8	12.0	42.0	29.9	12.1	37.9	25.1	12.8	37.4	24.7	12.6	35.4	22.9	12.5	41.5	28.8	12.8	42.3	29.4	12.8	37.7	24.2	13.5
**9.4**	24.6	13.9	10.7	17.8	12.1	5.7	24.6	13.6	11.0	35.7	21.5	14.2	30.3	17.3	12.9	28.9	16.8	12.1	28.2	15.9	12.3	31.6	19.8	11.8	34.4	21.7	12.8	30.0	16.8	13.2
**10.1**	30.3	20.4	9.9	26.9	19.1	7.8	30.6	23.8	6.8	40.2	31.9	8.3	35.7	26.4	9.4	36.3	26.1	10.3	36.3	26.2	10.0		29.8		40.6	34.8	5.8	34.2	24.8	9.3
**10.2**	27.8	18.6	9.3	25.0	18.2	6.8	28.9	22.5	6.4	39.3	30.0	9.3	33.6	24.7	8.9	34.7	24.9	9.9	34.9	24.9	10.1	39.7	30.7	8.9	40.5	33.6	6.9	32.9	23.7	9.1
**10.3**	28.1	18.4	9.7	24.2	17.7	6.5	28.9	22.6	6.3	40.8	30.2	10.6	33.3	24.7	8.6	34.4	25.0	9.5	34.7	24.8	9.9		36.7		39.2	32.2	7.1	32.5	23.8	8.7
**10.4**	28.1	18.7	9.4	25.3	17.6	7.6	28.8	21.8	7.0	39.1	29.8	9.3	34.1	24.8	9.3	34.9	24.8	10.1	34.7	24.8	9.9	39.0	33.8	5.2	38.7	31.2	7.5	32.3	23.2	9.1

Supplemental table 2

Overview of the complete data set including sociodemographic and epidemiologic characteristics from the questionnaire as well as results from RNA measurements including RNA quality and quantity measurements, 18S/16S measurements as well as gene expression results. Latter include the raw Ct values (threshold cycles) of potential housekeeping genes (n=10) analyzed for the 40 samples from 10 donors (four time points per donor). For each gene, the Ct values measured via qRT-PCR with cDNA without pre-amplification as well as after 14X pre-amplification is shown.
